# Supplementary material for: Stacking Model‐Based Classifiers for Dealing With Multiple Sets of Noisy Labels
Source: Biom J. 2025 Mar 12;67(2):e70042. doi: 10.1002/bimj.70042 (PMC11898607; doi:10.1002/bimj.70042)
Supplement: Supplementary file 1 — Supporting Information [file BIMJ-67-e70042-s001.zip › density-based-ensemble-model-main/competitors_real_data/netlab-master/netlab-master/help/rbfsetbf.htm]

Netlab Reference Manual rbfsetbf


# rbfsetbf

## Purpose

Set basis functions of RBF from data.

## Synopsis

```
net = rbfsetbf(net, options, x)
```

## Description

`net = rbfsetbf(net, options, x)` sets the basis functions of the
RBF network `net` so that they model the unconditional density of the
dataset `x`. This is done by training a GMM with spherical covariances
using `gmmem`. The `options` vector is passed to `gmmem`.
The widths of the functions are set by a call to `rbfsetfw`.

## See Also

`rbftrain`, `rbfsetfw`, `gmmem`

---

**Pages:**
Index


---

Copyright (c) Ian T Nabney (1996-9)
